# Comparison of rapid and same-day desensitization protocols in hypersensitivity reactions to platinum and taxanes: a retrospective cohort study

**DOI:** 10.3389/falgy.2026.1751512

**Published:** 2026-03-03

**Authors:** María Cruz Torres Górriz, Julián Borrás Cuartero, Paula Viedma Ayllón, Alfredo Sánchez Hernández, Carlos Vergara Hernández, Isabel Gil Viciano, Ernesto Enrique

**Affiliations:** 1 Allergy Department, Castellon University General Hospital, Castellon de la Plana, Spain; 2 Doctoral School. Jaume I University, Castellon de la Plana, Spain; 3 FISABIO Foundation Research Group, Valencia, Spain; 4 Allergy Department, Castellon Provincial Hospital Consortium, Castellon de la Plana, Spain; 5 Medical Oncology Departament, Castellon Provincial Hospital Consortium, Castellón, Spain; 6 FISABIO Foundation Statistical Studies Service, Valencia, Spain; 7 Radioimmunoassay Laboratory Service - Radiopharmacy, Castellon Provincial Hospital Consortium, Castellon de la Plana, Spain

**Keywords:** antineoplastic agents, breakthrough reactions, chemotherapy, rapid drug desensitization, same day desensitization

## Abstract

**Background:**

Rapid drug desensitization (RDD) allows first-line treatment to continue after a hypersensitivity reaction to the antineoplastic agent. There are different desensitization procedures, and none of them are free from breakthrough reactions (BTR).

**Objectives:**

The objective of the study is to evaluate and compare the efficacy of different desensitization procedures, considering the effect of a series of confounding variables, and to describe the characteristics of BTRs in different procedures.

**Methods:**

A retrospective, comparative review of medical records from patients who experienced hypersensitivity reactions to platinum- or taxane-based chemotherapy was conducted. Patients were categorized into two groups; rapid drug desensitization (RDD) and same-day desensitization (SDD), according to the intervention performed on the day of the initial reaction. Demographic data, drug, retreatment, cancer type, phenotype and severity of the initial reaction, allergy testing, number of desensitizations performed, outcome of desensitizations, and number, phenotype and severity of BTRs were recorded according to group.

**Results:**

In the RDD group, 406 desensitizations were analyzed in 76 subjects with a BTR rate of 24%. In the SDD group, 164 desensitizations were analyzed in 44 subjects with a BTR rate of 15%. A marginaleffects analysis using a Bayesian hierarchical model showed a 14.6-point reduction in the probability of BTR in the SDD group.

**Conclusion:**

This study confirms that desensitization procedures are safe and effective and allows us to conclude, based on the data and the model, that the SDD group has a lower probability of BTR than the RDD group.

## Introduction

Access to new antineoplastic therapies has improved survival rates in cancer patients. However, this inevitably leads to an increase in hypersensitivity reactions (HSR) to the drugs used (1). HSRs induced by antineoplastic drugs have a major impact, pose a significant risk to patient safety and complicate the management of cancer (2, 3).

The rate of HSR to antineoplastic drugs is at least 5%, although some reports describe rates as high as 46% for platinum agents (4–6). For taxanes, immediate HSR have been reported in up to 12% of cases (7).

The management of HSRs caused by antineoplastic drugs requires a multidisciplinary and comprehensive approach involving oncologists, hematologists, pharmacists, nurses and experienced allergists (4, 8). Rapid drug desensitization (RDD) is available to manage these reactions. In RDD, increasing the drug dose using a multi-step system blocks key mechanisms in mast cell activation (9, 10). RDD has been successfully used in hypersensitivity reactions to chemotherapeutic agents and monoclonal antibodies (mAbs) and allows continuation of first-line therapy (11–13) without compromising patient survival outcomes (14).

There are various desensitization protocols, with the 12-step protocol developed at Brigham and Women's Hospital being the most validated method (15, 16). However, new single-bag desensitization protocols have been developed with the aim of simplifying the process. This approach reduces the risk of drug spillage, prevents erroneous dilutions and shortens the duration of desensitization (17–21).

Recently, at the Castellon Provincial Hospital Consortium (CHPC), a modification of the simplified 1-bag, 10-step desensitization protocol was introduced, referred to as same-day desensitization (SDD) (22). This procedure consists of administering the culprit drug on the same day as the reaction, after achieving clinical stabilization of the patient. SDD uses the mechanisms widely defined for RDD and attempts to take advantage of the refractory state of degranulated mast cells, potentially related to post-anaphylactic mast cell anergy (PAMA) or “mast cell depletion syndrome” (10, 23). Data from the first patients undergoing SDD suggests that this approach is safe and effective, as it provides greater comfort and avoids loss of the treatment cycle in which the reaction occurred (22).

No desensitization protocol is entirely free from the risk of BTR during the procedure. Several publications have examined the risk factors for BTRs RDD for antineoplastic agents (24–29). However, no studies have compared or analyzed BTRs between RDD and SDD procedures.

This study presents data on the administration of platinum and taxanes using RDD and SDD. The main objective is to evaluate and compare the efficacy of RDD and SDD and to describe the characteristics of breakthrough reactions in them.

## Materials and methods

### Study design

This is a retrospective cohort study that included patients >18 years of age with cancer who had experienced HSR to platinum or taxanes at the Oncology Infusion Centre (OIC) of the CPHC over a period of three years.

Patients were divided into two groups according to the intervention performed by the allergist on the day of the index reaction.

The first group of patients, who underwent traditional RDD, was designated the deferred RDD group. These patients, after experiencing an initial HSR during chemotherapy, had their treatment suspended and were referred to the Allergy Department. Each patient was assessed by an allergist, who performed the relevant diagnostic tests and recommended desensitization when appropriate.

The second group, referred to as the SDD group, comprised patients who received their treatment via SDD after experiencing their initial reaction. Only patients whose initial reaction was witnessed, assessed, and treated by an allergist were eligible for inclusion in the SDD group. In addition, patients were required to be hemodynamically stable and to provide consent for the procedure. As with the deferred RDD group, patients in the SDD group were subsequently referred to the Allergy Department for further assessment and, when appropriate, continued their treatment regimen using RDD.

The study was approved by the Local Ethics Committee of the CPHC.

### Characteristics of the patients

Patient characteristics were assessed using data obtained from medical records. The information collected included age, sex, cancer type, culprit drug, current treatment regimen (first treatment or re-treatment), number of previous infusions prior to the index reaction and a clinical description of the reaction. The term “re-treatment” was used to refer to cases in which a patient received the same drug again after remaining disease-free for more than six months.

Patients in both groups met the following criteria: they had symptoms consistent with a HSR to platinum compounds or taxanes; they required continuation of the same therapeutic agent according to the oncologist's recommendation; they had completed at least one RDD procedure following the allergy assessment; and all had provided written informed consent.

Patients in the SDD group met the following additional criteria: the symptoms of an HSR to platinum compounds or taxanes had been witnessed and managed by the allergist *in situ* at the OIC; the patient had achieved clinical stabilization after the initial treatment; and desensitization with the culprit drug was carried out on the same day as the index reaction.

### Characteristics of the initial hypersensitivity reaction

The signs and symptoms of the index HSR were categorized as follows: cutaneous (erythema, pruritus, urticaria, angioedema and delayed maculopapular rash); cardiovascular (chest pain, tachycardia, syncope, hypotension and hypertension); respiratory (dyspnea, cough, wheezing and oxygen desaturation); laryngeal (dysphonia and dysphagia); gastrointestinal (nausea, vomiting, diarrhea and abdominal pain); neurological (dizziness, bradypsychia and altered level of consciousness); and other systemic manifestations (chills, fever, and lower back pain).

The index HSRs were classified as type I immediate reaction (IgE-mediated or non-IgE-mediated), cytokine release reaction (CRR), standard infusion reaction (SIR), mixed reaction and type IV reaction (30, 31).

SIRs are generally managed with symptomatic treatment, temporary interruption of the infusion and a reduction in the infusion rate. However, when SIRs recur despite premedication or when SIRs are severe, desensitization is preferred over simple re-exposure, as RDD enables a more gradual and controlled administration of the drug (31, 32).

The severity of immediate type I HSR was classified as mild (grade 1), moderate (grade 2) or severe (grade 3), according to the Brown classification system (33). The severity of SIRs and CRRs was assessed using the National Cancer Institute's Common Terminology Criteria for Adverse Events (34).

### *In vivo* and *in vitro* allergy testing

Skin tests were performed 13–20 days after the index HSR. Skin prick test (SPT) and intradermal test (IDT) were conducted according to previously published protocols (4, 35) and interpreted in accordance with the recommendations of the European Academy of Allergy and Clinical Immunology (EAACI) European Network on Drug Allergy (ENDA) (36). A positive SPT or a positive IDT at a 1:100 dilution was considered strongly positive (24).

Serum tryptase (ImmunoCAP™ Uppsala, Sweden), interleukin-6 (Quantikine® ELISA. Minneapolis, USA), a complete blood count and total immunoglobulin E (ImmunoCAP™ Uppsala, Sweden) were measured 90–120 min after the index HSR (37). Post-reaction tryptase was considered elevated if >11.4 ng/ml (38) or above (baseline tryptase × 1.2) + 2 ng/ml (39). Serum tryptase and interleukin-6 were also measured at baseline.

### Rapid drug desensitization protocol

All RDD protocols were individualized. Comorbidities, initial reaction severity, skin test results, serum biomarkers and the need for epinephrine were considered when selecting the protocol (15). Thirty minutes before desensitization, patient received standard premedication (oral ebastine 20 mg, iv famotidine 20 mg and sublingual diazepam 5 mg), in addition to drug-specific premedication. Beta-adrenergic blockers were discontinued 24 h before the procedure.

The final RDD outcome, the total number of desensitization cycles per patient, and the characteristics of any BTRs during desensitization were recorded.

### Same-day desensitization protocol

In patients in the SDD group, all SDDs were performed using a single bag. This protocol uses a single dilution of the drug in 10 steps, and it uses the same bag that was used to produce the index HSR (22). After completing SDD, all patients were referred to the Allergy Department for evaluation and subsequent indication of RDD.

The outcome of SDD, number and characteristics of BTR during SDD, total number of RDD performed in each patient after SDD, and number and clinical characteristics of BTRs produced in subsequent RDDs were recorded.

### Breakthrough reactions

Any event related to drug administration that occurred during desensitization or in the hours following it was considered a BTR. If a BTR occurred during desensitization, the infusion was interrupted, and the patient was treated. After clinical resolution, the protocol was resumed at a previous step or at the same step where the BTR had occurred. For subsequent desensitization procedures, additional premedication and/or intermediate steps were added before the step at which the BTR occurred, fluid therapy was added, the infusion rate was reduced. The phenotype and severity of BTRs were assessed in the same way as initial HSRs (30, 33, 34).

In both groups (deferred RDD and SDD), the overall BTR rate (number of procedures with BTR/number of procedures) and the successful procedure completion rate (number of procedures that reached the target dose/number of procedures) were calculated.

### Statistical analysis

A descriptive analysis of the data was performed according to the desensitization procedure (deferred RDD/SDD), number of drug cycles ≥10, positive SPT or IDT 1/100 result, significant tryptase after initial reaction, IgE levels >100 U/ml, and drug type (platinum/taxanes). The selection of adjustment variables in the analysis was performed according to the variables used in previous publications (22, 25–28). Quantitative variables were summarized using median and interquartile range, and qualitative variables using frequency and percentage.

To address the study objective, it was decided to model the occurrence of BTR in each of the sessions, considering their number and order. A hierarchical Bayesian model was proposed, consisting of a logistic regression with a random effect at the subject level, which was adjusted for sex, age, type of drug, re-treatment, number of cycles until initial HSR, positive result in SPT or IDT 1/100, post-reaction serum tryptase and IL-6 levels, and baseline total IgE levels, along with an effect for the number of sessions using a restricted cubic spline. Additionally, the model was adjusted for the severity grade of the index hypersensitivity reaction.

The brms package (40) was used for modelling with four parallel chains with 5,000 iterations each, discarding the first half. Convergence in each model was checked by reviewing the Gelman-Rubin statistic values and the effective number of simulations for each parameter. It was decided that if values greater than 1.1 and less than 100 (respectively) were obtained, the model would be re-evaluated. The fit of the model to the data was assessed by checking the predictive posterior distribution for the number of BTRs.

To facilitate the interpretation of the results, in addition to the OR for the type of desensitization adjusted for covariates, the Average Treatment Effect (ATE) was also estimated, without conditioning for random effects, using the *marginaleffects* package (41), so that negative values would indicate a lower probability of presenting BTR in the SDD group than in the deferred RDD group. Its posterior distribution was represented and the probability of it being less than zero was calculated. All credibility intervals were calculated at 95% and all analyses were performed in the R v4.5.1 statistical environment (42).

## Results

### Characteristics of the patients

The study included 76 patients in the deferred RDD group and 44 in the SDD group. Demographic and oncologic characteristics are summarized in Table 1. Overall, 77% of patients received platinum-based agents and 23% received taxanes. Carboplatin was the most frequent culprit drug in the deferred RDD group, whereas oxaliplatin predominated in the SDD group.

**Table 1:** Patient characteristics.

		Total number of patients	Deferred RDD	SDD
		*N* = 120	*N* = 76	*N* = 44
Sex	Male	39 (32.50%)	18 (23.68%)	21 (47.73%)
	Female	81 (67.50%)	58 (76.32%)	23 (52.27%)
Age	Median (Q1, Q3)	59.50 (50.50, 70.00)	62.00 (51.00, 71.00)	58.00 (50.50, 65.00)
Oncological diagnosis	Colon	35 (29.17%)	17 (22.37%)	18 (40.91%)
	Ovarian	27 (22.50%)	22 (28.95%)	5 (11.36%)
	Gastroesophageal	18 (15%)	5 (6.57%)	13 (29.54%)
	Breast	17 (14.17%)	14 (18.42%)	3 (6.82%)
	Cervicouterine	10 (8,33%)	10 (13.15%)	0
	Lung	6 (5%)	4 (5.26%)	2 (4.55%)
	Others	7 (5.83%)	4 (5.26%)	3 (6.81%)
Agent	Oxaliplatin	56 (46.67%)	23 (30.26%)	33 (75.00%)
	Carboplatin	33 (27.50%)	28 (36.84%)	5 (11.36%)
	Cisplatin	3 (2.50%)	3 (3.95%)	0 (0.00%)
	Paclitaxel	26 (21.67%)	21 (27.63%)	5 (11.36%)
	Docetaxel	2 (1.67%)	1 (1.32%)	1 (2.27%)
Re-treatment	Not	46 (38.33%)	31 (40.79%)	15 (34.09%)
	Yes	74 (61.67%)	45 (59.21%)	29 (65.91%)
N° of prior cycles before initial HSR	Median (Q1, Q3)	3.00 (2.00, 5.00)	2.00 (2.00, 4.00)	3.00 (2.00, 6.00)
	Min, max	1.00, 12.00	1.00, 12.00	1.00, 12.00

### Characteristics of the index hypersensitivity reaction

**Figure 1:**
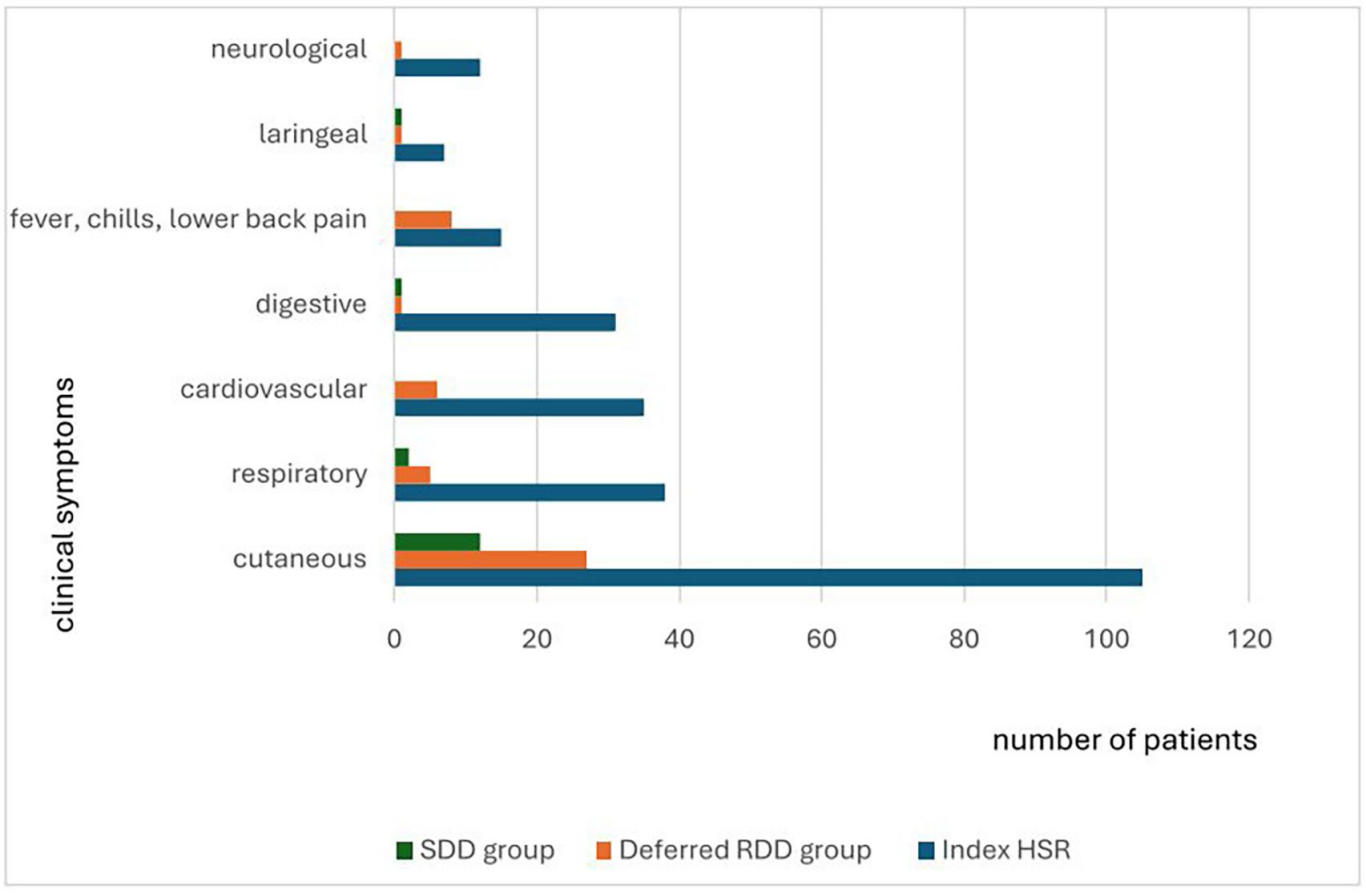
Index HSR symptoms, BTR symptoms in the deferred RDD group, and BTR symptoms in the SDD group.

The predominant index HSR phenotype in both groups was an immediate type I reaction. The characteristics of the index HSRs are summarized in Table 2 and symptoms are detailed in Figure 1.

**Table 2:** Characteristics of the initial HSRs.

		Total number of patients	Deferred RDD	SDD
		*N* = 120	*N* = 76	*N* = 44
Initial HSR severity	Mild	61 (50.83%)	30 (39.47%)	31 (70.45%)
	Moderate	40 (33.33%)	29 (38.16%)	11 (25.00%)
	Severe	19 (15.83%)	17 (22.37%)	2 (4.55%)
Initial HSR phenotype	Type I HSR	87 (72%)	52 (68.42%)	35 (79.54%)
	CRR	2 (2%)	1 (1%)	1 (2%)
	SIR	24 (20%)	18 (23.68%)	6 (13.63%)
	Type IV HSR	2 (2%)	2 (3%)	0
	Mixed reaction	5 (4%)	3 (4%)	2 (5%)
Use of adrenaline	Not	98 (81.66%)	58 (76.31%)	40 (90.90%)
	Yes	22 (18.33%)	18 (23.68%)	4 (9.09%)

Overall, index HSRs were mild in 51% of cases, moderate in 33%, and severe in 16%. All severe reactions were immediate. In the deferred RDD group, index HSRs were classified as mild in 39%, moderate in 38%, and severe in 22%. In contrast, in the SDD group, 70% of index HSRs were mild, 25% moderate, and 5% severe.

Intramuscular epinephrine was required in 24% of patients in the deferred RDD group compared with 9% in the SDD group.

### *In vivo* and *in vitro* allergy testing

Skin tests were performed in 84% of patients in the deferred RDD group and 93% in the SDD group. Skin test was not performed in a few patients due to high-dose steroids or antihistamines, histamine deficiency or weekly treatment regimen. The median interval between the index HSR and skin testing was 19 days. Approximately 80% of tested patients had positive results in both groups. In the deferred RDD group, carboplatin skin tests were positive in 92% of cases and oxaliplatin and cisplatin in 100%; paclitaxel tests were all negative, while the single patient with a docetaxel HSR had a positive test. In the SDD group, carboplatin tests were 100% positive, oxaliplatin 87%, and all taxane tests were negative. Tryptase was measured in 93% of patients, IL-6 in 83%, and total IgE in 72%. Results are shown in Table 3.

**Table 3:** Characteristics of allergy testing.

		Total number of patients	Deferred RDD	SDD
		*N* = 120	*N* = 76	*N* = 44
Skin test	Positive SPT	18 (17.14%)	12 (18.78%)	6 (14.63%)
	Positive IDT	64 (60.95%)	35 (54,68%)	29 (70,73%)
	Delayed positive	2 (1.90%)	2 (3.12%)	0
	Negative	21 (20.00%)	15 (23.43%)	6 (14.63%)
	Ur	15	12	3
Positive SPT or IDT 1/100		41 (39.05%)	25 (39.06%)	16 (39.02%)
Post-reaction tryptase (ng/ml)	Median (Q1, Q3)	6.95 (4.85, 10.35)	7.10 (5.00, 10.70)	6.80 (4.50, 9.10)
	Min, max	1.00, 41.70	2.50, 41.70	1.00, 39.60
	Ur	8	7	1
Basal tryptase (ng/ml)	Median (Q1, Q3)	6.15 (5.10, 7.60)	6.30 (5.20, 7.30)	5.70 (4.40, 7.80)
	Min, max	1.00, 16.20	1.00, 15.50	2.30, 16.20
	Ur	32	23	9
Significant tryptase	Not	54 (60.67%)	32 (59.26%)	22 (62.86%)
	Yes	35 (39.33%)	22 (40.74%)	13 (37.14%)
	Ur	31	22	9
Post-reaction IL-6 (pg/ml)	Median (Q1, Q3)	5.25 (2.45, 19.25)	9.90 (3.00, 21.00)	4.10 (2.20, 13.00)
	Min, max	0.50, 2,636.00	0.50, 2,636.00	1.00, 219.00
	Ur	20	17	3
Basal IL-6 (pg/ml)	Median (Q1, Q3)	5.40 (2.90, 12.00)	7.90 (3.10, 12.90)	4.50 (2.80, 11.80)
	Min, max	0.40, 328.70	0.40, 328.70	0.90, 55.00
	Ur	58	43	15
IgE total (U/ml)	Median (Q1, Q3)	125.50 (34.00, 384.00)	141.00 (35.00, 357.00)	106.50 (21.00, 645.00)
	Min, max	2.00, 5,000.00	2.00, 5,000.00	3.00, 2,843.00
	Ur	34	22	12
IgE total >100 U/ml		46 (53.49%)	30 (55.56%)	16 (50.00%)

Ur, unrealized.

### Description of breakthrough reactions in patients undergoing deferred RDD

In the deferred RDD group a total of 406 procedures were performed. Breakthrough reactions (BTRs) occurred in 36 patients, accounting for 97 events and yielding a BTR rate of 24% (97/406). Results are shown in Table 4. Cutaneous manifestations were the most common presenting symptom (87%) (Figure 1).

**Table 4:** RDDs and ocurrence of any BTR in both groups.

		Total number of patients	Deferred RDD	SDD
		*N* = 120	*N* = 76	*N* = 44
RDD	Number RDD	570	406	164
	Median (Q1, Q3)	4.00 (2.00, 6.00)	4.50 (2.50, 7.00)	3.00 (2.00, 5.00)
	Min, max	1.00, 27.00	1.00, 27.00	1.00, 11.00
Target dose achieved		567 (99.47%)	403 (99%)	164 (100%)
Occurrence of any BTR		48 (40.00%)	36 (47.37%)	12 (27.27%)

Sixty-nine percent of patients in the deferred RDD group experienced a BTR during their first desensitization procedure. Results are shown in Table 5.

**Table 5:** Patients with BTR.

		Number of patients with BTR deferred RDD group	Number of patients with BTR SDD group
		*N* = 36	*N* = 12
BTRs	Number BTRs	97	25
	Median (Q1, Q3)	2.00 (1.00, 3.00)	1.50 (1.00, 2.50)
	Min, max	1.00, 13.00	1.00, 7.00
BTR severity	Mild	28 (78%)	10 (83%)
	Moderate	4 (11%)	2 (17%)
	Severe	4 (11%)	0
BTR phenotype	Type I HSR	24	12
	CRR	4	0
	SIR	4	0
	Type IV HSR	2	0
	Mixed Reaction	1	0
	Type II HSR	1	0
Patiens with phenotype switch	Number of patients	8 (22%)	1 (8%)
	Type I HSR	0	1
	CRR	3	0
	SIR	2	0
	Type IV HSR	1	0
	Mixed reaction	1	0
	Type II HSR	1	0
Agent	Platines	32 (89%)	12 (100%)
	Taxanes	4 (11%)	0
BTR during the first RDD		25 (69%)	11 (91.66%)

Regarding severity, 78% of BTRs were mild, 11% moderate, and 11% severe. Compared with the index HSR, 89% of patients had BTRs of equal or lesser severity, while 11% (4 patients, 5 BTRs) experienced more severe reactions.

In three of these five BTRs, the target drug did not reach. However, in the two BTRs that were more severe than the index reaction, the target dose was successfully administered through desensitization. One patient who reached the target dose did not undergo further RDDs due to the development of a non-desensitizable phenotype: a type II cytotoxic reaction known as oxaliplatin immune syndrome.

Among patients in the deferred RDD group who experienced BTRs, 22% (8 patients) exhibited a change in phenotype compared with their initial HSR. More than half of these patients shifted to the CRR/SIR phenotype. One patient developed a mildly delayed reaction, another a severe type II reaction and a further patient shifted to a mixed reaction.

### Description of breakthrough reactions in patients undergoing SDD

In this group, three patients experienced BTRs during desensitization on the same day as the index reaction. All were type I and presented exclusively with cutaneous symptoms. None of these three patients developed BTRs in subsequent RDD.

In the SDD group, 164 desensitizations were performed. Twenty-five BTRs occurred in twelve patients, yielding a BTR rate of 15%. Results are shown in Table 4. All BTRs in this group occurred with platinum-based drugs and cutaneous symptoms were observed in all cases (Figure 1).

Of the patients in the SDD group who developed a BTR, 92% (11 patients) had it during their first RDD. Results are shown in Table 5.

Regarding severity, 83% of BTRs were mild and 17% moderate. No severe BTRs were recorded. All RDDs performed in patients in the SDD group reached the target dose of the drug. When compared with the severity of the index HSR, 83% of patients with BTRs had the same phenotype and a severity equal to or lower than that of the index HSR. However, 17% of patients (2 patients) with BTRs showed the same phenotype but with greater severity. None of these reactions required treatment with intramuscular epinephrine.

Only one patient in the SDD group exhibited a change in phenotype after the BTR. This patient, who initially had a mixed reaction, developed an isolated type I reaction during subsequent desensitization.

### Desensitization outcome: deferred RDD group vs. SDD group

**Figure 2:**
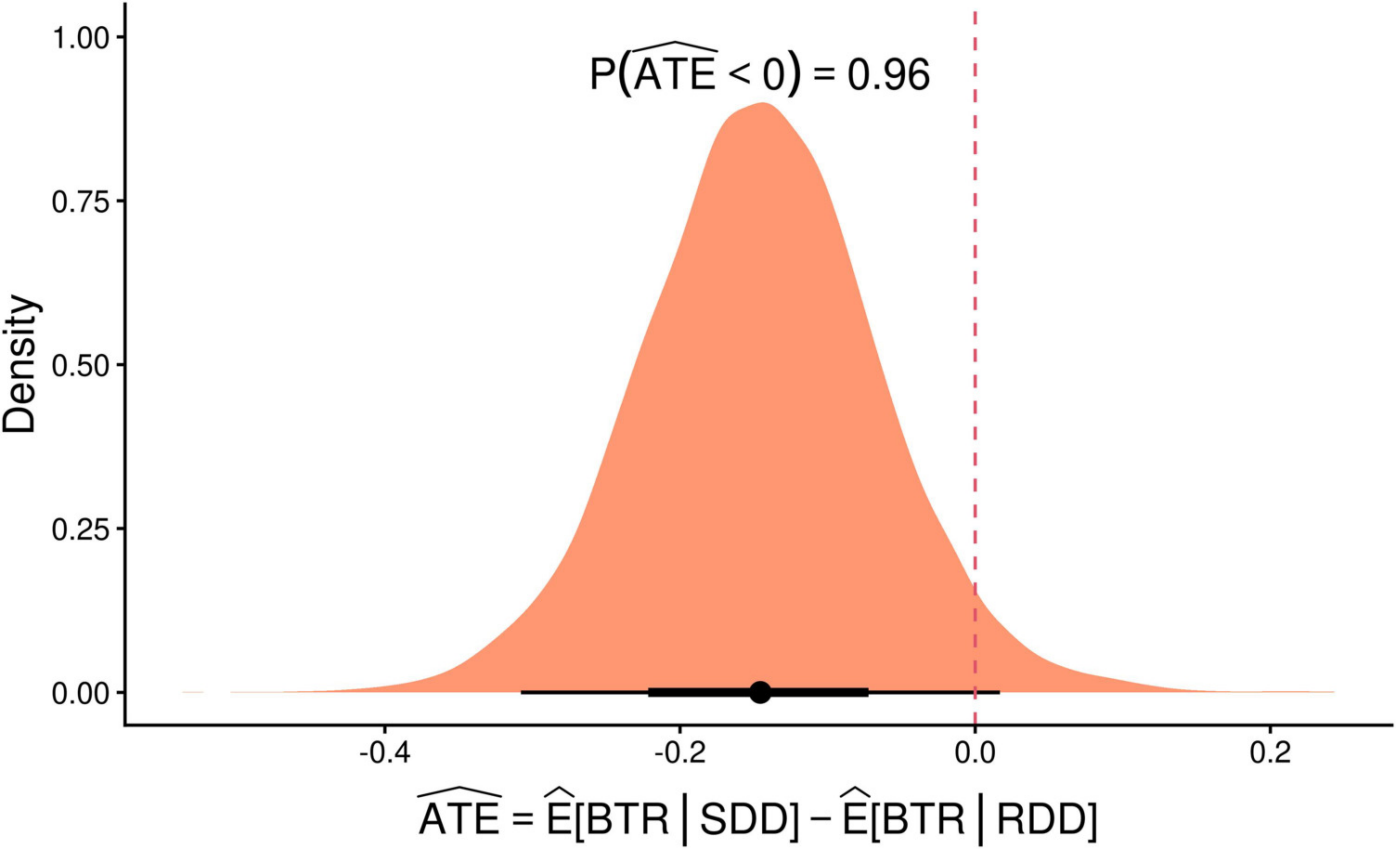
Density plot of the posterior distribution of the average treatment effect (ATE).

In this study, a hierarchical Bayesian model was proposed, and a multivariate analysis was performed in which the parameters of all models converged without incident. The predictive posterior distribution for the number of BTRs was consistent with the observed data (Table 6). After performing a *marginaleffect* analysis, without conditioning for random effects, the point estimate was −0.146 (95% CrI: −0.308; 0.017) with a probability that the effect was less than zero of 0.96. In the density plot of the posterior distribution of the ATE in this model, an estimate below 0 indicates a lower probability of BTR, while a value above 0 suggests a higher probability of BTR. As shown in Figure 2, nearly the entire density curve lies below 0, indicating that the SDD group has a lower probability of BTR during the procedures compared with the deferred RDD group. This represents an estimated absolute reduction of 14.6 percentage points in the probability of BTR for SDD compared with deferred RDD.

**Table 6:** Crude analysis adjusted for confounding variables between BTR development and type of desensitization.

	ORc	CrI 95%	ORa	CrI 95%
Type of procedure				
Deferred RDD	1.00	–	1.000	–
SDD	0.31	0.06, 1.52	0.069	0.002, 1.307
Sex				
Male	1.00	–	1.000	–
Female	1.69	0.34, 9.22	2.230	0.154, 42.80
Age	1.05	0.99, 1.13	0.965	0.852, 1.088
Agent				
Taxanes	1.00	–	1.000	–
Platins	21.7	2.95, 260	12.70	0.092, 5,583
Re-treatment				
Not	1.00	–	1.000	–
Yes	1.37	0.29, 6.93	5.375	0.138, 363.6
Cycle number of the reaction	1.62	1.25, 2.19	2.338	1.277, 5.075
Grade of the initial reaction				
Mild	1.00	–	1.000	–
Moderate	1.28	0.24, 6.66	0.949	0.044, 20.32
Severe	0.55	0.05, 4.89	1.304	0.016, 116.8
Positive skin test by SPT or IDT 1:100				
Not	1.00	–	1.000	–
Yes	23.1	4.91, 160	40.58	3.302, 1,108
Post-reaction tryptase (ng/ml)	1.00	0.90, 1.11	0.941	0.758, 1.168
Baseline total IgE (U/ml))	0.88	0.56, 1.37		
Post-reacción IL-6 (pg/ml)	1.23	0.72, 2.14	1.782	0.785, 4.757

Ora, adjusted odds ratio; ORc, crude odds ratio; CrI, credible interval.

## Discussion

This study assesses and compares BTRs occurring during two desensitization procedures (RDD and SDD) to platinum and taxane agents. It also examines whether intervention with SDD is associated with a decrease probability of BTRs in subsequent procedures.

The overall BTR rate across all procedures was 21%. The BTR rate in the deferred RDD group was 24% and 15% in the SDD group. Several publications report data on BTR rates, which are like those observed in this study. Castells et al. reported that 27% of desensitization procedures were associated with mild BTRs (15). Caiado et al. found a BTR rate of 9.6% during RDD (24). Gorgulu et al. observed a rate of 24% (25), whereas Kim et al. reported a BTR rate of 26% (27). The variability in BTR rates among different studies may be explained by the range of drugs involved and the differing protocols employed. Nevertheless, recent publications comparing single-bag and multi-bag approaches have not shown significant differences in safety or efficacy (20, 21).

In this study, most BTRs occurred during the first desensitization, consistent with other publications (16, 25) in which the risk of BTR decreases with subsequent exposures, particularly in type I HSR. However, BTRs can still occur after several RDD cycles have been tolerated without incident, emphasizing the need for strict monitoring during desensitization regardless of the number of uneventful procedures. Some studies suggest that desensitization induces temporary drug tolerance (12, 43) while more recent research indicates the possibility of achieving long-term tolerance or immunological anergy following repeated RDD, mediated by the induction of regulatory cytokines, predominantly interleukin-10, and the appearance of regulatory T cells producing IL-35 (44).

In this study, as previously reported in the literature (24, 25), the majority of BTRs in both groups were of equal or less severity than the initial reaction. However, we observed that up to 5% of BTRs in the deferred RDD group occurred with greater severity than the initial reaction. Additionally, one publication has reported a rate of BTRs with greater severity than the initial reaction of up to 18% (28). Therefore, any desensitization procedure should be considered high-risk and must always be conducted under the supervision of experienced allergists.

In addition to potential changes in severity during a BTR, there is also the possibility of a change in reaction phenotype. In this study, 22% of patients in the deferred RDD group experienced a change in phenotype, whereas 8% of patients with BTRs in the SDD group showed such a change. Phenotype switching, previously described with oxaliplatin, supports the notion that RDD blocks IgE-dependent mast cell activation mechanisms while potentially triggering the activation of other immune cells and pathways (45, 46).

Several publications have analyzed risk factors for BTRs during desensitization, and most agree that identifying these factors improves the management of hypersensitivity reactions to antineoplastic agents (13). Severe initial HSR, history of drug allergy and prior exposure to high drug doses have been shown to increase the risk of moderate-to-severe BTRs during platinum desensitization (26–28, 47). Other risk factors analyzed include positive skin tests (13, 25, 28, 29), atopy (13) and a total IgE level >100 U/ml (24).

A limitation of this study is its retrospective design. Although the statistical analysis was adjusted for variables that showed baseline differences between groups, it cannot be ruled out that the results are the consequence of selection bias. Among its strengths is the high rate of determination of *in vitro* biomarkers. These biomarkers assist the allergist in performing precise endophenotyping, allowing for safer management of desensitization procedures.

One might assume that the lower proportion of initially severe reactions in the SDD group could introduce bias when comparing the two groups. However, this variable was controlled in the statistical analysis. It is also noteworthy that oxaliplatin has been linked to a higher rate of BTRs (46), and in this study it was the culprit drug in all patients who experienced a BTR in the SDD group.

Although both procedures showed good efficacy and safety, consistent with previous reports (22, 24–29), early intervention with SDD may reduce the likelihood of BTRs. This effect could be related to the shorter interval between the index HSR and SDD, potentially offering a protective benefit. However, some authors (29) have suggested that a short interval between the index reaction and the first RDD may itself constitute a risk factor for BTRs. Therefore, alternative explanations should be considered, such as the possibility that early intervention following the index HSR could induce immunological changes that reduce the likelihood of BTRs in subsequent RDDs.

SDD has proven effective for managing HSRs to platinum- and taxane-based chemotherapy. Beyond underscoring the allergist's key role, this study suggests an added benefit: patients receiving early SDD show improved tolerance in subsequent RDD cycles.

## Data Availability

The original contributions presented in the study are included in the article/Supplementary Material, further inquiries can be directed to the corresponding author.
